# Impact of a ketogenic diet intervention during radiotherapy on body composition: III—final results of the KETOCOMP study for breast cancer patients

**DOI:** 10.1186/s13058-020-01331-5

**Published:** 2020-08-20

**Authors:** Rainer J. Klement, Colin E. Champ, Ulrike Kämmerer, Petra S. Koebrunner, Kelley Krage, Gabriele Schäfer, M. Weigel, Reinhart A. Sweeney

**Affiliations:** 1 Department of Radiation Oncology, Leopoldina Hospital, Robert-Koch-Straße 10, 97422 Schweinfurt, Germany; 2 Department of Radiation Oncology, Duke University Medical Center, Durham, NC USA; 3 Department of Obstetrics and Gynaecology, University Hospital of Würzburg, Würzburg, Germany; 4 Department of Obstetrics and Gynaecology, Leopoldina Hospital, Breast Cancer Centre, Schweinfurt, Germany

**Keywords:** Bioimpedance analysis, Diet, Ketone bodies, Nutrition

## Abstract

**Background:**

Obesity and low muscle mass are associated with worse outcomes of breast cancer patients. We conducted a controlled trial to study the impact of a ketogenic diet (KD) based on natural foods versus an unspecified standard diet (SD) on body composition in breast cancer patients undergoing radiotherapy.

**Methods:**

Patients with non-metastasized breast cancer were allocated to either the KD (*N* = 32) or the SD (*N* = 31) during radiotherapy. Body composition was measured weekly by bioimpedance analysis. Blood parameters and quality of life were assessed before, during, and at the end of radiotherapy.

**Results:**

A total of 29 KD and 30 SD patients completed the study. During radiotherapy, mean and median fasting BHB concentrations in the KD group were 0.72 and 0.49 mmol/l (range 0.06–4.9) which was significantly higher than those in the SD group (*p* < 2.2 × 10^−16^). There was a very small and insignificant increase in body weight and fat mass in the SD group, as well as a decrease of fat free mass. In contrast, patients in the KD group lost body weight and fat free and skeletal muscle mass quickly after diet onset, which for the most part was related to water losses. The KD did not cause further substantial changes in fat free or skeletal muscle mass, but was associated with a gradual decrease of 0.4 kg body weight and fat mass per week (*p* < 0.0001). The KD significantly decreased free T3 levels by 0.06 pg/ml/week (*p* = 6.3 × 10^−5^). Global quality of life remained stable in the SD group but increased in the KD group from a score of 66.7 to 75.0 (*p* = 0.20).

**Conclusions:**

In breast cancer patients undergoing curative radiotherapy, a KD based on natural foods is feasible. After initial water losses, the KD tends to reduce body weight and fat mass while preserving fat free and skeletal muscle mass.

**Trial registration:**

ClinicalTrials.gov identifier: NCT02516501, registered on August 06, 2015.

## Background

Breast cancer is the most frequent cancer in women and in 2018 was the leading cause of cancer-related death in women worldwide [1]. In Germany, breast cancer is the third leading cause of cancer-related deaths and poses a relevant economic burden on the healthcare system [2]. As a result, there is burgeoning interest in researching modifiable factors that causally impact the treatment outcome and prognosis of breast cancer patients. Besides well-known prognostic factors such as age, stage of disease, HER2-Neu expression, and estrogen and progesterone receptor status, factors relating to the cancer patient’s metabolism such as obesity [3, 4], sarcopenia [5, 6], insulin levels [7, 8], and chronic hyperglycemia [9–11] have been shown to possess a prognostic role. Evidence for a causal influence of these metabolic factors comes from preclinical data showing that breast cancer cells are stimulated by insulin [12] and certain adipokines [13] and are vulnerable to glucose restriction [14, 15]. Unfortunately, a large proportion of newly diagnosed breast cancer patients exhibit high fasting blood glucose levels [16], obesity, and low muscle mass [5, 6]. These phenomena may be exacerbated during radio- and chemotherapy, worsening the health and fitness of patients. In fact, many women tend to gain weight during therapy, which by itself has been associated with negative treatment outcomes [17]. Thus, research into interventions that improve the body composition and metabolic health of women is necessary as it could potentially improve the prognosis of these patients. Lifestyle modifications are of particular interest since they allow patients to take self-responsibility during their treatment.

Along these lines, a large percentage of women are interested in receiving recommendations for a “healthy diet” during treatment. For example, out of 37 breast cancer patients undergoing curative radiotherapy in a Swiss study, 70% were extremely interested in receiving dietary advice [18]. However, current dietary guidelines may be suboptimal for halting weight gain and improving body composition and metabolic health of breast cancer patients during and after their therapy [17]. Furthermore, current recommendations vary widely [19], further illustrating the need for evidence-based guidelines supported by dietary research. A high-fat ketogenic diet (KD) has been proposed by some authors, as it appears to not only promote weight loss comparable to a low-fat diet, but also favorably impact metabolic parameters associated with cancer treatment outcomes [17, 20, 21]. A KD induces a state of physiological ketosis, which is defined as β-hydroxybutyrate (β-OHB) levels ≥ 0.5 mmol/l [22].

To test the hypothesis that a KD during radiotherapy can positively influence body composition and metabolic parameters, we have launched a prospective, non-randomized, controlled phase I clinical trial, the KETOCOMP study [23]. This study has been approved by the ethics committee of the Bavarian Medical Association (Landesaerztekammer Bayern) and registered under ClinicalTrials.gov identifier NCT02516501. In a first interim analysis, we have reported favorable effects on body composition of seven breast cancer, eight rectal cancer, and five head and neck cancer patients following a KD during radiotherapy compared to control patients on a standard diet (SD) [24]. Here, we report the final results of the KETOCOMP study for the subgroup of breast cancer patients.

## Materials and methods

### Patient accrual and characteristics

The original study protocol was designed for three cohorts of breast, rectal, and head and neck cancer patients [23]. Here, we only deal with the breast cancer cohort. The study protocol stipulated that patients should be enrolled into two intervention groups: intervention group 1 should receive a ketogenic breakfast consisting of up to 250 ml of a ketogenic drink containing 20 g medium-chain triglycerides per 100 ml (betaquik®, vitaflo, Bad Homburg, Germany) plus essential amino acids in form of the “Master Amino Acid Pattern” supplement (MAP, recently re-branded as MyAMINO, dr. reinwald healthcare gmbh + co kg, Altdorf, Germany); intervention group 2 should follow a whole food KD supplemented with MAP [23]. However, owing to the observation that more than half of rectal and head and neck cancer patients in the KETOCOMP study were unable to tolerate the maximum target dose of betaquik®, intervention group 1 was closed before any breast cancer patients were recruited into it. The protocol was subsequently amended to shift focus to intervention group 2 which will hereafter be referred to as the KD group, with the target number of patients in intervention group 1 transferred to the KD group (15 breast cancer patients). Furthermore, due to great interest among breast cancer patients in the study, the protocol was amended to enroll another 15 patients into the KD group but without additionally receiving 10 g MAP on radiation days, so that any putative effect of the essential amino acids contained in MAP on body composition could in principle be separated from that of the KD. The target number for the control group consuming an unspecified SD was also raised to a total of 30 patients.

According to the study protocol [23], eligible patients included women between 18 and 75 years of age with non-metastatic breast cancer referred to our clinic for curative radiotherapy. Exclusion criteria were as follows: Karnofsky index < 70, body mass index (BMI) < 18 kg/m^2^, metallic implants (because of possible interference with body composition measurements), pregnancy, cognitive impairment, inability to speak or understand German, and metabolic defects posing a contraindication against consuming a KD. No financial compensation was offered, and patients were free to exit the study at any time.

**Fig. 1:**
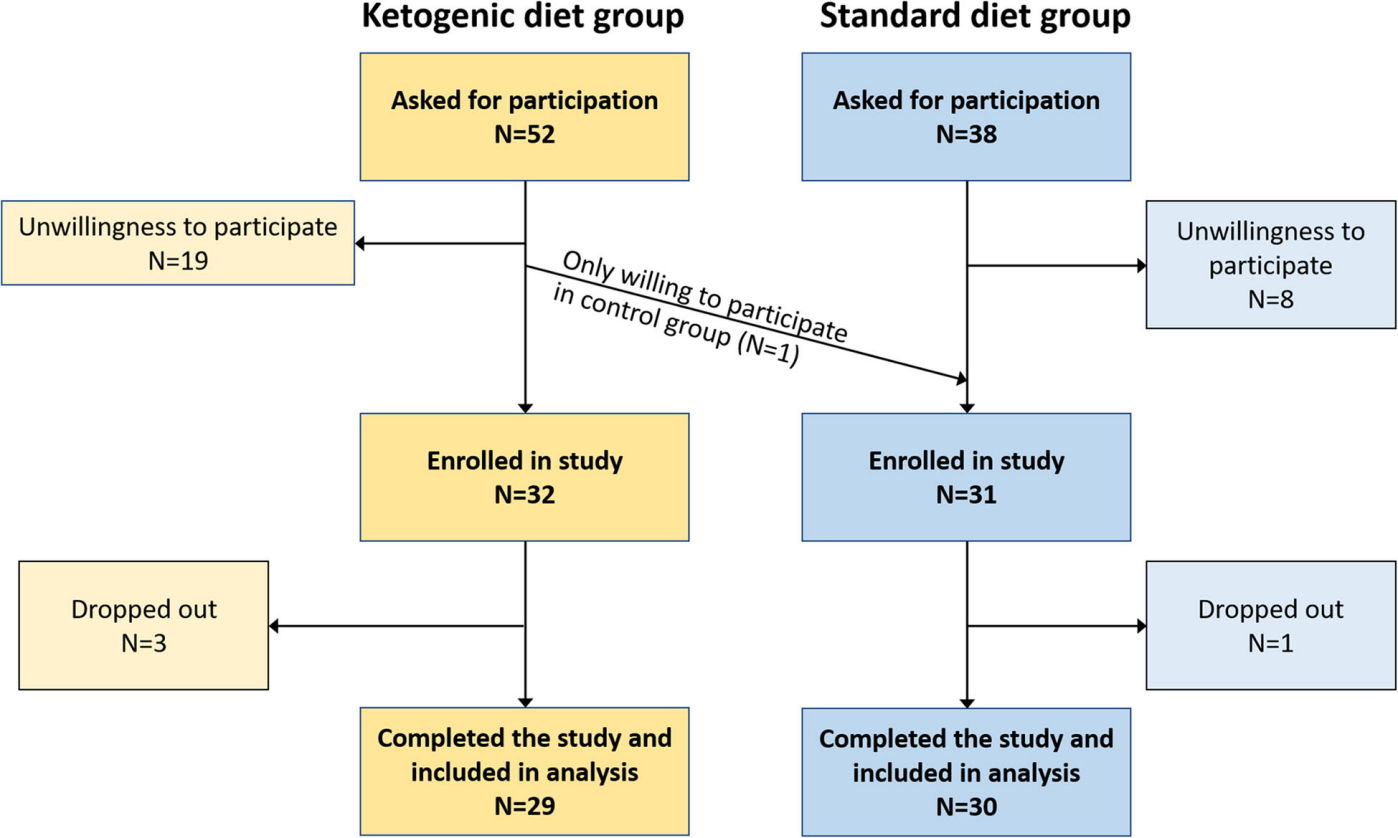
Flowchart of patient recruitment. Reasons for dropout in the KD group were non-compliance with the dietary advice and stress

In general, patients that presented for radiotherapy in the Leopoldina Hospital Schweinfurt and fulfilled the inclusion criteria were asked at first consultation by their treating oncologist if they would be willing to participate. Patient allocation into the intervention and control groups was performed consecutively in the following manner: We first recruited five patients into the KD group in order to gain first experiences with patients’ compliance to the diet and feasibility of the protocol; one of them was a patient we already described in some detail in a previous publication who had served as a test patient of the trial protocol in August 2014 [25], 1 year before the trial had been registered. In a second step, we then aimed to fill the SD group with consecutive patients, followed by filling the rest of the KD group in a third step. The reason for this recruitment pattern was to mitigate patient self-selection bias towards one diet or the other (through inter-patient discussions, e.g., in the waiting room). However, if patients were only willing to engage in one of the dietary groups due to strong preferences, this was allowable as the research team felt this would not compromise the goal of the study, and would support compliance with the diet. Thus, patients supposed to enter the KD group, in accordance with the recruitment design, but not wanting to eat a KD were offered to participate in the SD group. This offer was accepted by one patient. Figure 1 shows details of the recruitment process.

### Measurements

In general, patients presented fasted and with an empty bladder on the same morning they received their radiotherapy planning computed tomography for initial (baseline) measurements, approximately 1 week prior to beginning radiotherapy. Baseline measurements consisted of (i) weight and bioimpedance analysis (BIA) on a calibrated seca 515/514 medical Body Composition Analyzer (mBCA; seca Deutschland, Hamburg, Germany), (ii) the validated EORTC QLC-C30 questionnaire version 3.0 together with the BR23 module, and (iii) blood draw with subsequent analysis in the hospital laboratory (including blood cell counts, metabolic parameters, liver enzymes, insulin, IGF-1, free T3 and T4 [23]). BIA and weighing were repeated weekly during radiotherapy. In addition, laboratory blood analysis and completion of the quality of life (QoL) questionnaire were repeated once during and in the final week of radiotherapy.

At the final examination, each patient completed a short non-validated questionnaire asking about the type of her diet (a multiple choice question intended as a compliance check and including categories such as “Ketogenic,” “Balanced,” “Low carb,” and “Vegetarian,” among others), supplement and drug intake, and type and amount of exercise during radiotherapy. Patients in the KD group were also asked about their subjective well-being while on the diet and their future dietary plans.

The primary outcome measures for this study were as follows:

- Dropout rate in the KD group
- Changes from baseline to the final week of radiotherapy in body composition parameters: body weight (BW), fat free mass (FFM), fat mass (FM = BW − FFM), skeletal muscle mass (SMM), extracellular water (ECW), total body water (TBW), and intracellular water (ICW = TBW − ECW)
- Changes in bioimpedance phase angle at 50 kHz (PA)

### Dietary intervention

In general, patients in the KD group were counseled about the principles and practice of a KD at the day of first consultation with their treating oncologist (about 5 min), and received a thorough dietary consultation by a registered dietician with experience in implementing KDs on the day of baseline measurements. In addition, they received handouts including food choices and cooking recipes and had the option to borrow recipe books and a popular book on the KD and cancer [26]. MCT oil was recommended (Kanso 100% MCT, Dr. Schär AG, Burgstall, Italy) and given to the patients for free. Our guidelines suggested replacing carbohydrates with fat, consuming 75–80% calories from fat, and limiting carbohydrates to 50 g per day and 10 g per meal. The consumption of an ad libitum whole food KD was promoted. High-quality protein of animal origin and micronutrient-dense foods were emphasized, as was the avoidance of industrial and processed foods (with the exception of MCT oil), vegetable oils (except virgin coconut and olive oil), grains, and legumes. Dairy products were suggested only in moderation and preferably in the form of butter, cheese, and fermented products. Patients in the KD group were requested to start the KD after baseline measurements, but at least 2 days prior to their first radiotherapy treatment. To check compliance with the KD, patients had to fill out a food diary for 2 days, measure urinary acetoacetate concentration daily by means of urinary ketone strips (Ketostix, Bayer Vital GmbH, Germany), and were regularly asked about the implementation of the diet. In addition, blood ketones and glucose were checked at least once weekly after the BIA measurement using the FreeStyle Precision device (Abbott Diabetes Care Ltd., Range Road, Witney, UK). For 15 patients, the KD was supplemented with 10 g MAP on radiation days that patients consumed directly after irradiation.

Patients in the SD group received no specific dietary advice. However, patients could ask for dietary counseling, which was requested by four patients. These individuals received standard recommendations according to the German Nutrition Society (DGE) [27], which promote consuming mostly unrefined foods of plant origin (in particular whole grains, vegetables, and fruits) and limiting fats to 30–35% daily energy intake, with an emphasis on reducing fats from animal origin.

### Statistical analysis

Longitudinal body composition data of the *N* patients were analyzed using linear mixed effects models with the intercept and slope of the variable *t* (time since start of radiotherapy) as random effects varying by the individual patient. Let *y*_*ij*_, *i* = 1, …, *n*_*j*_, denote the *i*th measurement on patient *j* = 1, …, *N* at time *t*_*i*_ during radiotherapy. Our basic model (*M*1) predicted an individual body composition measurement *y*_*ij*_ based on time, group (0 = SD; 1 = KD), their interaction, and the corresponding baseline body composition measure *y*_0*j*_:

$$ M1:{y}_{ij}=\left({\beta}_0+{U}_j\right)+\left({\beta}_1+{W}_j\right)\times {t}_i+{\beta}_2\times {\mathrm{KD}}_j+{\beta}_3\times {\mathrm{KD}}_j\times {t}_i+{\beta}_4\times {y}_{0j}+{\epsilon}_{ij}, $$

$$ {U}_j\sim N\left(0,{\sigma}_U^2\right),j=1,\dots, N. $$

$$ {W}_j\sim N\left(0,{\sigma}_W^2\right) $$

1 $$ {\epsilon}_{ij}\sim N\left(0,{\sigma}_{\epsilon}^2\right),i=1,\dots, {n}_j $$

The average time trends of patients in the SD and KD groups are therefore given by *β*_1_ and *β*_1_ + *β*_3_, respectively.

The second model was similar to model *M*1, but additionally including the baseline BMI which could in principle predict how likely a person is to lose or gain additional BW, FM, and/or FFM:

2 $$ M2:{y}_{ij}=\left({\beta}_0+{U}_j\right)+\left({\beta}_1+{W}_j\right)\times {t}_i+{\beta}_2\times {\mathrm{KD}}_j+{\beta}_3\times {\mathrm{KD}}_j\times {t}_i+{\beta}_4\times {y}_{0j}+{\beta}_5\times {\mathrm{BMI}}_{0j}+{\epsilon}_{ij}, $$

Since body composition may also be influenced by the anabolic stimulus of essential amino acids (in particular leucine), we tested a model including an interaction between intake of MAP (0 = no, 1 = yes) and time:

3 $$ M3:{y}_{ij}=\left({\beta}_0+{U}_j\right)+\left({\beta}_1+{W}_j\right)\times {t}_i+{\beta}_2\times {\mathrm{KD}}_j+{\beta}_3\times {\mathrm{KD}}_j\times {t}_i+{\beta}_4\times {y}_{0j}+{\beta}_5\times {\mathrm{MAP}}_j\times {t}_i+{\epsilon}_{i\mathrm{j}}. $$

The fourth model included age as a possible factor influencing body composition changes:

4 $$ M4:{y}_{ij}=\left({\beta}_0+{U}_j\right)+\left({\beta}_1+{W}_j\right)\times {t}_i+{\beta}_2\times {\mathrm{KD}}_j+{\beta}_3\times {\mathrm{KD}}_j\times {t}_i+{\beta}_4\times {y}_{0j}+{\beta}_5\times {\mathrm{Age}}_j+{\epsilon}_{ij}. $$

Since the irradiated volume (planning target volume) could correlate with inflammation and fatigue, we hypothesized a possible influence of this variable on body composition:

5 $$ M5:{y}_{ij}=\left({\beta}_0+{U}_j\right)+\left({\beta}_1+{W}_j\right)\times {t}_i+{\beta}_2\times {\mathrm{KD}}_j+{\beta}_3\times {\mathrm{KD}}_j\times {t}_i+{\beta}_4\times {y}_{0j}+{\beta}_5\times {\mathrm{PTV}}_j+{\epsilon}_{ij}. $$

Finally, a global model was built including all of the above variables:

6 $$ M6:{y}_{ij}=\left({\beta}_0+{U}_j\right)+\left({\beta}_1+{W}_j\right)\times {t}_i+{\beta}_2\times {\mathrm{KD}}_j+{\beta}_3\times {\mathrm{KD}}_j\times {t}_i+{\beta}_4\times {y}_{0j}+{\beta}_5\times {\mathrm{BMI}}_{0j}+{\beta}_6\times {\mathrm{MAP}}_j\times {t}_i+{\beta}_7\times {\mathrm{Age}}_j+{\beta}_8\times {\mathrm{PTV}}_j+{\epsilon}_{ij}. $$

Models were compared using the second-order bias-corrected Akaike Information Criterion (AICc). Differences in AICc measure the strength of evidence for one model over another, and models differing more than 8 from the model with the lowest AICc were considered to have substantially less evidential support from the data [28]. The general adequacy of the model set {*M*1, …*M*6} was judged based on the conditional and marginal *R*^2^ values of the “global model” *M*6 [29]; for their calculation, see Nakagawa et al. [30]. Also, the intervention group variable was omitted from the best model and its AICc was re-calculated in order to judge the effect of the KD intervention.

To ease interpretability of the regression coefficients, the covariates age and BMI were scaled to have mean zero and standard deviation 10 years or 10 kg/m^2^, respectively. To take into account the intra-individual prediction errors in body composition data, we generated for each subject 1000 Monte Carlo simulations of a BIA measurement. Briefly, a new body composition measurement was simulated by drawing a random number with mean located at the actual measurement value and standard deviation equal to 100 g for BW or the root mean square error estimates derived by Bosy-Westphal et al., respectively, which are 1.91 kg for FFM, 1.2 kg for SMM, 0.79 l for ECW, and 1.34 l for TBW [31, 32]. For each simulated dataset, a new mixed effects model was fit, their regression coefficients and standard errors were averaged, and corresponding *p* values were calculated.

The analysis of longitudinal QoL and blood parameter data was also performed using linear mixed effects models adjusted for age and BMI.

Differences between continuous and categorical variables were assessed using the Mann-Whitney-Wilcoxon and Fisher’s exact test, respectively. Because *p* values overstate the evidence against the null hypothesis [33, 34], we decided to speak of “significant” findings only if *p* values < 0.005 were obtained, in line with a recent proposal by statisticians [35]. All analyses were carried out in R, version 3.5.0 with the software package lme4 for linear mixed effects modeling.

## Results

### General results

We had separated the three waves of recruitment (five patients into KD followed by SD followed by rest of KD group) by longer breaks, so that (after testing the protocol on one patient in 2014 who was included in the KD group) the first patient was enrolled into the study in August 2016, and the last patient in March 2020. The recruitment process resulted in an approximately balanced distribution of patient characteristics with the exception of a higher, yet clinically insignificant, β-OHB concentration in the KD group (Table 1). The latter difference was likely due to a number of patients in the KD group having already reduced carbohydrate consumption after being asked to participate in the study, despite our request to maintain their normal diet until the day of baseline measurements. The percentage of potentially eligible patients intended for the KD group declining their participation (35%) was larger than that of patients intended for the SD group (Fig. 1). During the intervention, there were three study dropouts in the KD group due to non-compliance with the KD prescription, but not as a result of any side effects. With this 9% dropout rate in the KD group, the intervention was judged as feasible. One patient in the SD group ended the study after 1 week due to stress from the weekly measurements. Therefore, the following analyses are based on 29 patients in the KD and 30 patients in the SD group. The median study duration (time from start of radiotherapy until final measurement) was 35 days (range 19–47 days) in the KD and 35 days (18–43 days) in the SD group (*p* = 0.217).

**Table 1:** Baseline characteristics of the intervention and control groups

Parameter	KD group (*n* = 29)	SD group (*n* = 30)	*p* value
Age [years]	52 (29–78)	53 (25–68)	0.785
Body weight [kg]	74.2 (47.5–120)	70.0 (48.1–124.2)	0.336
BMI [kg/m^2^]	28.3 (19.9–45.2)	25.0 (18.8–43.0)	0.157
Fat mass [kg]	30.3 (13.3–65.6)	26.5 (12.0–69.9)	0.208
Fat free mass [kg]	44.2 (30.8–54.4)	43.0 (31.5–54.3)	0.596
Skeletal muscle mass [kg]	20.5 (11.5–26.2)	19.6 (13.2–23.8)	0.370
50 kHz phase angle [°]	4.98 (4.22–5.67)	4.65 (3.72–5.88)	0.050
Estrogen receptor status			0.423
*Positive*	27 (93.1%)	24 (80.0%)	
*Negative*	2 (6.9%)	5 (16.7%)	
*Unknown*	0	1 (3.3%)	
Progesterone receptor status			0.530
*Positive*	24 (82.8%)	21 (70%)	
*Negative*	5 (17.2%)	8 (26.7%)	
*Unknown*	0	1 (3.3%)	
HER2/neu status			0.790
*Positive*	11 (37.9%)	13 (43.3%)	
*Negative*	18 (62.1%)	16 (53.3%)	
*Unknown*	0	1 (3.3%)	
Affected breast			0.435
Left	18 (62.1%)	15 (50%)	
Right	11 (37.9%)	15 (50%)	
Smoking status			0.265
No	15	10	
Active	4	8	
Formerly	10	12	
Tobacco consumption [pack years]	0 (0–60)	3 (0–30)	0.249
Radiotherapy fractions	28 (16–31)	28 (16–31)	0.449
PTV [cm^3^]	1092 (398–2091)	999 (345–2475)	0.383
Glucose [mg/dl]	99 (82–176)	96 (81–113)	0.197
β-OHB [mmol/l]	0.11 (0.01–0.45)	0.06 (0.02–0.29)	0.0188
Insulin [mU/l]	7.7 (2–45.6)	8.1 (2.5–27.2)	0.928
IGF-1 [ng/ml]	197 (45–364)	212 (116–348)	0.901
T3 [pg/ml]	3.13 (2.63–4.13)	3.16 (2.04–4.5)	0.823
Global quality of life score	66.7 (33.3–100)	66.7 (0–100)	0.981

Continuous and categorical variables are presented as median (range) and frequencies, respectively. *BMI* body mass index, *IGF-1* insulin-like growth factor 1, *KD* ketogenic diet, *PTV* planning target volume, *SD* standard diet

### Dietary adherence

During radiotherapy, mean and median fasting BHB concentrations in the KD group were 0.72 and 0.49 mmol/l (range 0.06–4.9), respectively, which were significantly higher than those in the SD group (mean 0.13, median 0.06, range 0.02–2.59 mmol/l, *p* < 2.2 × 10^−16^). There were two BHB measurements exceeding 0.5 mmol (0.53 and 2.59 mmol/l) in the SD group, both in the same patient who, however, confirmed that she was not on a special diet or fasting regimen. Nevertheless, a loss of 2.1 kg BW (1.5 kg FFM) during radiotherapy and decrease in insulin levels (from 3.7 to 1.9 mU/l) suggest that this patient inadvertently had restricted calories. A total of 9 postprandial BHB measurements were also taken randomly in the KD group and revealed median levels of 0.9 mmol (0.3–1.9 mmol/l). Only two patients did not manage to have at least one BHB measurement ≥ 0.5 mmol/l. Three patients had to be excluded from the KD group because interviews and food diaries revealed non-compliance with the principles of a KD; all other patients appeared compliant based on 2-day food diaries and urinary ketone measurements.

### Influence on body composition

**Fig. 2:**
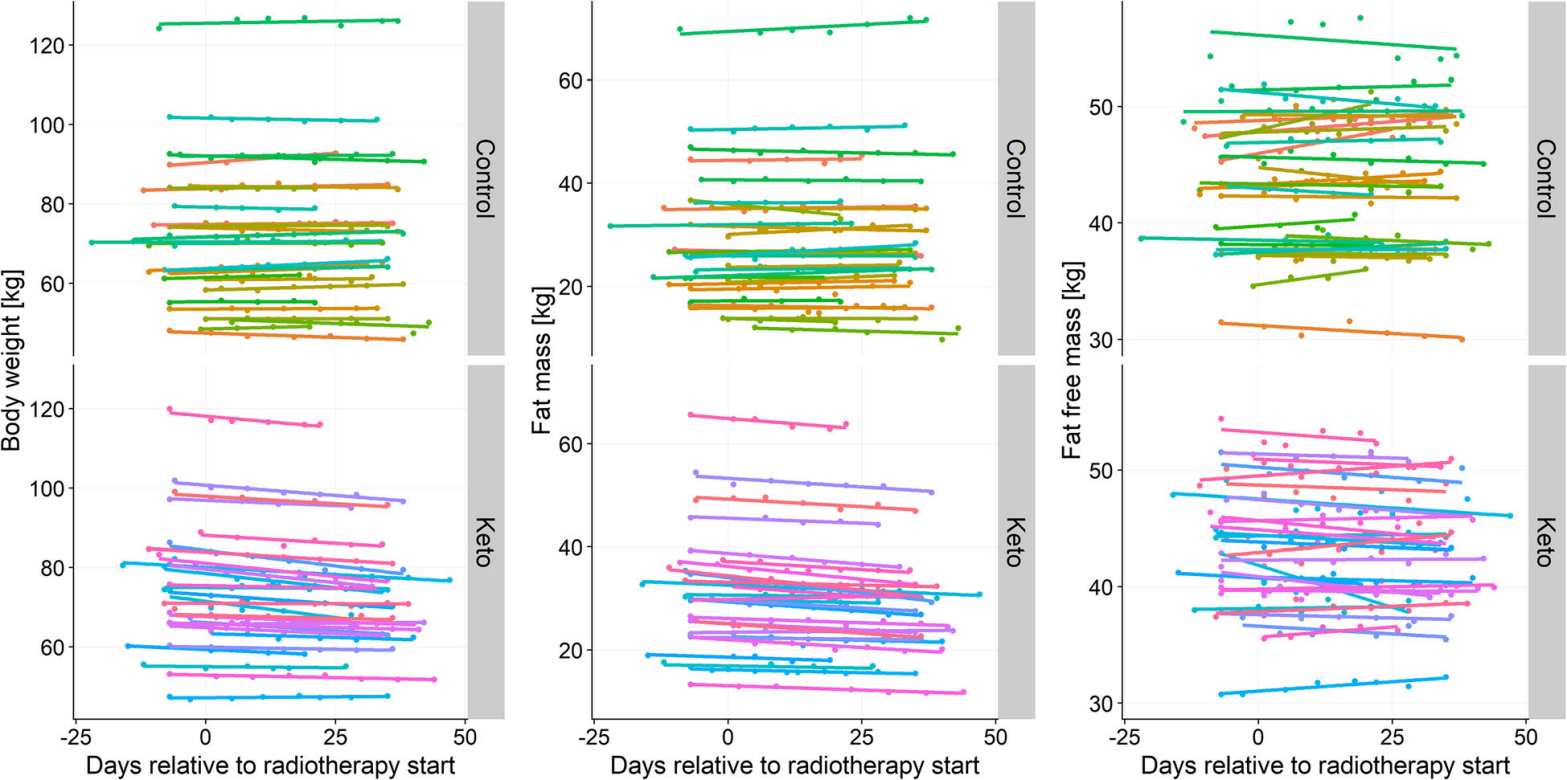
Individual changes in body weight, fat mass, and fat free mass in the KD (Keto) and SD (Control) groups. The lines are linear regression lines, each fitted to an individual patient’s longitudinal body composition data

The gradual BW, FM, and FFM changes are plotted in Fig. 2. The lines represent simple linear regression models fitted to each patient’s longitudinal data. It is visible that in the KD group, there was a uniform pattern of gradual decreases in BW and FM, while FFM changes were more heterogeneous.

In order to model the average trends in body composition changes, we deployed linear mixed effects models as described by Eqs. (1)–(6) above. Supplementary Table 1 contains the AICc values for the different models. The best model having the smallest AICc value is highlighted in bold. The last column shows that omitting the KD variable from the best model resulted in a substantially worse fit, because AICc increased by ≳ 8, except when modeling changes in ICW where the AICc difference between the best model and the best model without the KD effect was only 6.1. The best models were obtained with the inclusion of initial BMI for modeling changes in BW; inclusion of a MAP × time interaction for modeling changes in FM; inclusion of age for modeling changes in FFM, SMM, ICW, and TBW; and inclusion of all confounders for modeling changes in PA; for ECW changes, the simple model without additional confounders was the best one (Supplementary Table 1). For all body composition parameters, the data did not provide substantially strong evidence for the best model against the full model (all AICc differences < 6.1). We therefore decided to fit the full models to the longitudinal body composition data and show their regression coefficients in Tables 2 and 3. Fitting the full models also has the advantage that the direction of effects for every covariate can be seen.

**Table 2:** Regression coefficients and *R*^2^ values of the full models fitted to the body composition changes

Covariate	Body weight		Fat mass		Fat free mass		Skeletal muscle mass		50 kHz phase angle	
	Coefficient	*p* value	Coefficient	*p* value	Coefficient	*p* value	Coefficient	*p* value	Coefficient	*p* value
Time	0.04 ± 0.05 kg/week	0.40	0.08 ± 0.05 kg/week	0.10	− 0.05 ± 0.04 kg/week	0.22	− 0.01 ± 0.03 kg/week	0.72	0.02 ± 0.01°/week	0.012
KD: yes	**− 1.56 ± 0.25 kg**	**6.9 × 10**^**−10**^	− 0.35 ± 0.15 kg	0.020	**− 1.23 ± 0.27 kg**	**3.4 × 10**^**−6**^	**− 0.71 ± 0.19 kg**	**1.9 × 10**^**−4**^	0.17 ± 0.08°	0.038
Time × KD	**− 0.42 ± 0.08 kg/week**	**4.4 × 10**^**−7**^	**− 0.48 ± 0.08 kg/week**	**1.5 × 10**^**−9**^	0.09 ± 0.07 kg/week	0.19	− 0.02 ± 0.04 kg/week	0.64	**− 0.04 ± 0.01°/week**	**0.0041**
Baseline BMI	− 0.49 ± 0.67 kg/10 kg/m^2^	0.47	0.29 ± 0.53 kg/10 kg/m^2^	0.58	0.13 ± 0.39 kg/10 kg/m^2^	0.73	0.55 ± 0.26 kg/10 kg/m^2^	0.033	0.28 ± 0.10°/10 kg/m^2^	0.0064
Time × MAP	0.12 ± 0.09 kg/week	0.19	0.15 ± 0.09 kg/week	0.086	− 0.05 ± 0.08 kg/week	0.52	− 0.02 ± 0.05 kg/week	0.60	− 0.01 ± 0.01°/week	0.54
Age	− 0.22 ± 0.11 kg/10 years	0.041	0.09 ± 0.07 kg/10 years	0.19	− 0.26 ± 0.11 kg/10 years	0.025	− 0.20 ± 0.08 kg/10 years	0.012	**− 0.13 ± 0.04°/10 years**	**3.7 × 10**^**−4**^
PTV	− 0.15 ± 0.23 kg/500 ccm	0.51	− 0.19 ± 0.13 kg/500 ccm	0.16	0.16 ± 0.25 kg/500 ccm	0.51	0.004 ± 0.16 kg/500 ccm	0.98	− 0.12 ± 0.07°/500 ccm	0.061
Conditional *R*^2^	0.999		0.999		0.991		0.986		0.941	
Marginal *R*^2^	0.992		0.993		0.956		0.942		0.678	

Regression coefficient estimates are given with their standard error and associated *p* value. Note that the average time trends for the SD and KD groups are given by the regression coefficients corresponding to “Time” and “Time” + “Time × KD,” respectively. *BMI* body mass index, *KD* ketogenic diet, *MAP* Master Amino Acid Pattern supplement, *PTV* planning target volume

**Table 3:** Regression coefficients and *R*^*2*^ values of the full models fitted to the water composition changes

Covariate	Extracellular water		Intracellular water		Total body water	
	Coefficient	*p* value	Coefficient	*p* value	Coefficient	*p* value
Time	− 0.05 ± 0.02 l/week	0.024	− 0.01 ± 0.02 l/week	0.76	− 0.05 ± 0.03 l/week	0.13
KD: yes	**− 0.6 ± 0.2 l**	**3.2 × 10**^**−5**^	**− 0.5 ± 0.2 l**	**0.0014**	**− 1.2 ± 0.2 l**	**1.2 × 10**^**−7**^
Time × KD	0.09 ± 0.03 l/week	0.011	− 0.001 ± 0.03 l/week	0.97	0.08 ± 0.06 l/week	0.16
Baseline BMI	− 0.1 ± 0.2 l/10 kg/m^2^	0.57	0.4 ± 0.2 l/10 kg/m^2^	0.045	0.2 ± 0.3 l/10 kg/m^2^	0.49
Time × MAP	− 0.004 ± 0.03 l	0.91	− 0.03 ± 0.04 l	0.47	− 0.02 ± 0.06 l	0.69
Age	0.05 ± 0.06 l/10 years	0.38	**− 0.2 ± 0.1 l/10 years**	**0.0017**	− 0.14 ± 0.09 l/10 years	0.099
PTV	0.2 ± 0.1 l/500 ccm	0.15	0.01 ± 0.13 l/500 ccm	0.96	0.1 ± 0.2 l/500 ccm	0.48
Conditional *R*^2^	0.982		0.981		0.988	
Marginal *R*^2^	0.927		0.922		0.953	

Regression coefficient estimates are given with their standard error and associated *p* value. Note that the average time trends for the SD and KD groups are given by the regression coefficients corresponding to “Time” and “Time” + “Time × KD,” respectively. *BMI* body mass index, *KD* ketogenic diet, *MAP* Master Amino Acid Pattern supplement, *PTV* planning target volume

As can be read from Tables 2 and 3, the KD was associated with a general, time-independent, and statistically significant reduction in BW, FFM, SMM, ECW, ICW, and TBW. Furthermore, there were significant gradual decreases in BW (− 0.4 kg/week) and FM (− 0.4 kg/week), while there was no significant time trend for FFM, SMM, and water compartments. In the SD group, the average time trends indicated very small and non-significant increases in BW, FM, and PA and decreases in FFM and SMM.

Another significant association that was expected was the inverse association of age with FFM, SMM, and PA, the latter association being highly significant. Contrary to expectation, intake of the MAP supplement was not associated with increases in FFM, SMM, or PA, but with a gradual, non-significant (*p* = 0.086) FM gain of 150 g/week (Table 2).

The full model fits were also used in the Monte Carlo simulations carried out in order to study the influence of intra-individual prediction errors. The resulting average regression coefficients and *p* values are given in Supplementary Table 2. They are similar to those from the analysis using the actual data (Tables 2 and 3), but have larger uncertainties. However, there was now a significant association of baseline BMI with FM which was not found when analyzing the original data. These simulations confirm the overall picture of the KD leading to a gradual decline of BW and FM, but not FFM and SMM.

**Fig. 3:**
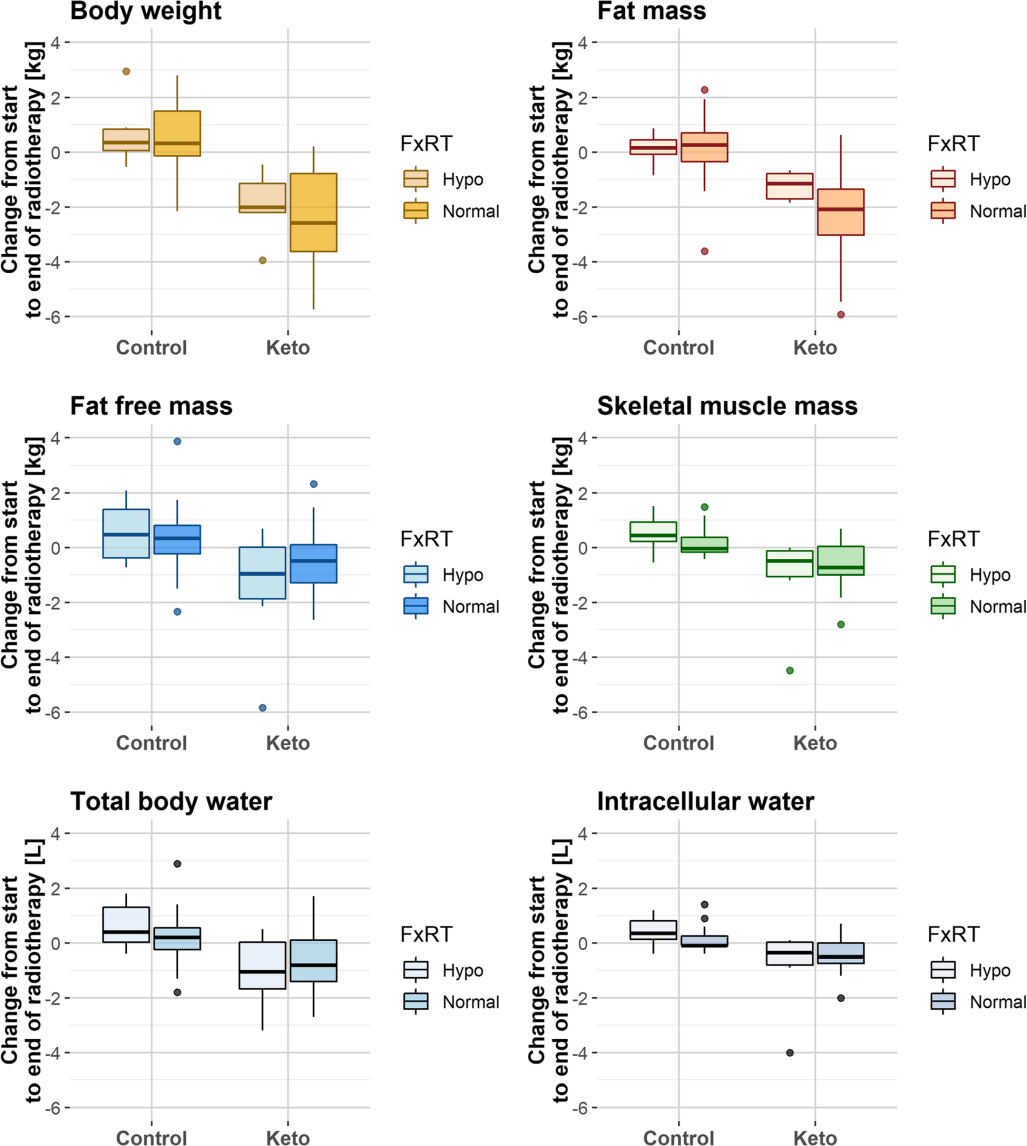
Absolute changes in body composition for patients receiving different fractionated radiotherapy (FxRT): hypofractionation (16-19 fractions) or normofractionation (25–31 fractions)

We also conducted an explorative, not a priori planned analysis of absolute body composition changes according to group and radiotherapy fractionation scheme, because the latter determined the time on the diet. The absolute values are given in Table 4 and plotted in Fig. 3. A total of 12 patients (6/6 in the KD/SD group) had been treated with hypofractionation (16 or 19 fractions, respectively). In those, the SD group experienced a mean weight gain of 0.7 ± 1.2 (standard deviation) kg, while the KD group lost 2.7 ± 1.2 kg, the difference being statistically significant (*p* = 0.0043). A total of 47 patients had been treated with normofractionation (26–31 fractions). Of those, patients in the SD group gained 0.4 ± 1.3 kg BW, 0.1 ± 1.3 kg FM, 0.3 ± 1.2 kg FFM, and 0.1 ± 0.5 kg SMM, while patients in the KD group lost on average 2.9 ± 2.2 kg BW, 2.3 ± 1.7 kg FM, 0.6 ± 1.3 kg FFM, and 0.6 ± 0.8 kg SMM. However, in the KD group, 0.8 ± 1.2 kg FFM and 0.4 ± 0.9 kg SMM decrease had already occurred between baseline and the second measurement; the changes between the second and final measurement amounted to a gain of 0.1 ± 0.8 kg FFM and a loss of 0.3 ± 0.7 kg SMM. Furthermore, as can be seen in Fig. 3, the amounts of FFM and SMM lost in the KD group were closely paralleled by the losses of TBW and ICW, respectively. Indeed, losses of FFM showed a strong correlation with losses of TBW (Spearman’s *ρ* = 0.97, *p* < 2.2 × 10^−16^) and losses of SMM with losses of ICW (*ρ* = 0.96, *p* < 2.2 × 10^−16^) in the KD group.

**Table 4:** Absolute changes in body composition parameters, β-hydroxybutyrate, and metabolic hormones

Fractionation	Hypofractionation			Normofractionation		
Group	KD (*N* = 6)	SD (N = 6)	*p* value	KD (*N* = 23)	SD (*N* = 24*)	*p* value
Δ Body weight [kg]	− 2.7 ± 1.2	0.7 ± 1.2	**0.0043**	− 2.9 ± 2.2	0.4 ± 1.3	**7.9 × 10**^**−7**^
Δ Fat mass [kg]	− 1.2 ± 0.5	0.1 ± 0.6	0.0087	− 2.3 ± 1.7	0.1 ± 1.3	**9.1 × 10**^**−6**^
Δ Fat free mass [kg]	− 1.5 ± 2.4	0.6 ± 1.1	0.065	− 0.6 ± 1.3	0.3 ± 1.2	0.030
Δ Skeletal muscle mass [kg]	− 1.1 ± 1.7	0.5 ± 0.7	0.015	− 0.6 ± 0.8	0.1 ± 0.5	**0.0012**
Δ Phase angle [°]	− 0.25 ± 0.82	0.06 ± 0.18	0.75	0.06 ± 0.29	0.05 ± 0.24	1
Δ Extracellular water [l]	− 0.2 ± 0.7	0.2 ± 0.3	0.52	− 0.3 ± 0.6	0.1 ± 0.7	0.033
Δ Intracellular water [l]	− 0.9 ± 1.6	0.4 ± 0.6	0.025	**− 0.5 ± 0.9**	**0.1 ± 0.4**	**0.0040**
Δ Total body water [l]	− 1.1 ± 1.4	0.6 ± 0.9	0.065	− 0.7 ± 1.1	0.2 ± 0.9	0.010
Δ β-hydroxybutyrate [mmol/l]	0.2 ± 0.2	− 0.03 ± 0.04	0.044	0.6 ± 0.7	0.1 ± 0.5	**8.2 × 10**^**−7**^
Δ Insulin	2.3 ± 7.5	− 1.9 ± 1.7	0.297	− 2.1 ± 5.9	− 0.5 ± 3.3	0.412
Δ IGF-1	− 23.2 ± 45.8	− 28.2 ± 30.9	0.294	− 22.9 ± 61.5	− 9.8 ± 40.9	0.116
Δ T3	− 0.25 ± 0.25	− 0.07 ± 0.05	0.310	− 0.48 ± 0.29	− 0.09 ± 0.38	**0.0012**

Body composition changes (Δ) from baseline to final measurement according to fractionation schedule which determined the study length. Significant differences between the KD and SD groups are highlighted in bold

*In the SD group, there was one patient with missing estimates at the final measurement except for body weight and phase angle because a wrong height had been inserted into the BIA device. This patient is only used for the calculations of body weight and phase angle changes

### Safety and quality of life

No grade > 1 diet-related adverse events were reported by the patients. When patients in the KD group were asked about their prospective diet after radiotherapy, 23 patients stated they would continue on a low-carbohydrate diet and five continued on a KD diet; only one patient decided to switch back to her pre-study diet.

Initial and final global QoL scores were available for 26 and 30 patients from the KD and SD group, respectively. Initially patients in the KD and SD group displayed a very similar QoL (Table 1). However, at the end of the intervention, global QoL had increased in the KD group only (from a score of 66.7 to 75.0), but this was not statistically significant (*p* = 0.202). The difference in QoL at the end of the intervention between the KD and SD groups was also not statistically significant (*p* = 0.162).

### Influence on blood parameters

We hypothesized that the KD would have an influence on insulin and IGF-1 and T3 hormone levels [36, 37]. The absolute changes in these hormones are given in Table 4, while Supplementary Table 3 shows the results of age- and BMI-adjusted linear mixed effects regression analysis of the hormone data. Insulin and IGF-1 decreased in both groups, but slightly more in the KD group. However, the KD caused a highly significant average drop in T3 levels of 0.06 pg/ml/week. A higher BMI was significantly associated with higher insulin levels (+ 4.5 mU/l per 10 kg/m^2^, *p* = 3.9 × 10^−4^), and age was highly significantly correlated with lower IGF-1 levels (− 37 ng/ml per 10 years, *p* = 9.5 × 10^−11^).

## Discussion

In this work, we illustrate that an individualized KD for breast cancer patients undergoing curative radiotherapy was safe and led to significant changes in body composition compared to an unspecified SD. The KD induced weight loss, primarily by reducing ICW and FM. These results are in line with, and thus confirm, our interim analysis that already revealed these effects [24].

Obesity is known to correlate with the risk of breast cancer development and recurrence, and several adipose tissue-mediated mechanisms including immune dysregulation, chronic systemic inflammation, and elevated growth factors may account for a causal correlation [38, 39]. While undesired weight loss is common in a broad variety of cancer entities, the opposite is generally true for breast cancer patients who tend to gain more weight during and after treatment [17]. Some data suggest that being overweight (BMI > 25 kg/m^2^) and having excess adipose tissue mass and low muscle mass at breast cancer diagnosis are associated with lower disease-free and overall survival [40]. Furthermore, there is evidence suggesting that post-diagnosis weight gain adversely affects disease-free survival [41]. For this reason, we focused on body composition outcomes in addition to BMI and BW, hoping to identify a practical diet regimen that would prevent both weight gain and muscle loss.

Comparing the body composition changes of patients on a KD and unspecified SD during radiotherapy (over a median of 35 days for both groups), we found that the KD intervention was associated with an average reduction of both BW and FM by 0.4 kg/week (Table 2). Additionally, body composition measurements revealed that FFM and SMM generally dropped in the KD group (Fig. 3 and Table 4), in parallel with decreases of TBW and ICW. Most of these changes had occurred already at the second measurement, soon after initiation of the KD, and in contrast to BW and FM, there was no indication for any further gradual decrease of FFM, SMM, TBW, or ICW. We believe that this general and rapid effect of the KD on FFM and SMM could be accounted for by a rapid loss of TBW and ICW occurring within the first days after diet onset. Accordingly, the decline of FFM and SMM was tightly correlated with that of TBW and ICW. Such water loss is a natural and expected consequence of the KD-induced depletion of glycogen stores, since each gram of muscle or liver glycogen is stored with at least 3 g of water [42–44]. In addition, because insulin increases the reabsorption of sodium in the kidneys [45], KDs typically exert a rapid diuretic effect by lowering average insulin levels which could have contributed to water loss in the KD group.

We therefore conclude that the KD initially induced a rapid reduction of BW through water loss, followed by a further gradual decrease consisting almost entirely of FM reduction, while FFM was preserved. In contrast, the average time trend for the SD group only indicated very small gains in BW and FM (0.04 and 0.08 kg/week, respectively) and very small decreases in FFM (− 0.05 kg/week). Given the large standard errors of these estimates (Table 2), we conclude that the SD did not change body composition substantially during radiotherapy. These results are consistent with the few studies that have investigated longitudinal body composition changes in early stage breast cancer patients undergoing radiotherapy. In a prospective Brazilian study including 23 breast cancer patients, there was a small, but insignificant increase of BW of 0.4 kg during radiotherapy, and no significant change in phase angle. In a Swiss study of 37 breast cancer patients, BW and FM increased from 64.4 ± 8.5 kg and 23.3 ± 5.8 kg, respectively, at radiotherapy start to 64.9 ± 8.6 kg and 23.7 ± 5.8 kg at radiotherapy end, while FFM remained stable [18]. This average weight gain of 0.5 kg is similar to our data, in which patients on a SD gained 0.7 ± 1.2 and 0.4 ± 1.3 kg, respectively, after hypo- and normofractionated radiotherapy (Table 4). In the cited study by Genton et al. [18], patients also reported increased fatigue levels associated with less appetite and less physical activity, and other data confirm a small, but significant decrease in activity levels during radiotherapy [46]. Thus, decreased physical activity during radiotherapy could be one explanation for the small gradual weight gain generally observed in patients who do not receive a dietary intervention, although we did not track changes in physical activity of our patients to further confirm this hypothesis. However, we asked each patient about the amount and type of physical activity they engaged in during radiotherapy. With a total of seven patients in each group who did exercise for at least 3 h per week, there was no apparent difference in physical activity between both groups that would confound the effects on body composition associated with the KD.

Our results are in line with a study in women with endometrial or ovarian cancer in which a KD based on whole foods resulted in significantly greater reductions of total, android, and visceral fat mass over 12 weeks than a diet recommended by the American Cancer Society, whereas total lean mass remained constant on both diets [36].

Of interest is the finding that intake of 10 g MAP to each radiotherapy fraction was not associated with any increase in FFM or SMM. An explanation for the general lack of beneficial effects of the MAP supplement on body composition might be that patients consumed sufficient amounts of high-quality protein as emphasized by our dietary guidelines, so that the additional amino acids were not utilized for further muscle anabolism.

In addition to body composition, we looked at breast cancer patients’ hormone profiles before, during, and at the end of the study to identify any significant or desired changes from adhering to a KD. Insulin and IGF-1 are of particular oncological interest, since both are known as growth factors for tumor cells [39] and might provide a mechanistic link between the increased risk of obesity and breast cancer incidence and recurrence. Overall, the KD had no significant effects on insulin and IGF-1 levels compared to the SD, although both hormones gradually decreased to a slightly greater extent in the KD group (Supplementary Table 3). This decrease may have been more pronounced with longer diet duration as is indicated by the greater absolute change in insulin and IGF-1 in patients who received 26–31 fractions compared to only 16 or 19 fractions (Table 4). The relatively short diet duration is a limitation of this study, but was determined by the duration of radiotherapy.

There was also a significant drop in T3 hormone by 0.06 ± 0.01 pg/ml/week (*p* = 6.3 × 10^−5^) associated with the KD. This association remained stable when restricting the analysis to 22 KD and 21 SD patients who did not take thyroxin medication (coefficient − 0.06 ± 0.02 pg/ml/week, *p* = 5.0 × 10^−4^). This is an interesting, yet not unexpected, finding for weight loss and KDs [37].

This analysis focused on the primary study outcome of body composition changes. Another potential benefit of a KD during radiotherapy that is mainly supported by preclinical data could be a synergistic anti-tumor effect mediated through the ketogenic state and its effect on a variety of molecular signaling pathways [47–51]. A randomized controlled trial conducted by Khodabakhshi and colleagues found an overall survival benefit (*p* = 0.046) for 25 locally advanced or metastatic breast cancer patients eating a KD during neoadjuvant chemotherapy compared to 18 control patients who received chemotherapy without dietary intervention [52]. With a median follow-up of 4.4 months (range, 0.7–65.1), our data are currently not adequate to address the question about the efficacy of the KD as a complementary, synergistic adjunct to radiotherapy. With longer follow-up data that we will collect, we may be able to address this question in the future.

The major limitation of this study is that patients self-selected to enter the KD group and that the KD food composition was not standardized, but highly individual. Although we tried to account for self-selection bias to some extent by our consecutive recruitment scheme and by adjusting for several putative confounders in the linear regression model analysis, there might have been some residual confounders that remained unaccounted for. Apart from macronutrient prescriptions, our KD protocol was not standardized, but rather provided a framework for designing personalized diets that would allow each individual to achieve and remain in nutritional ketosis. This lowers the degree to which the study outcome truly measures the effect of the intervention on the outcome in the study population (internal validity) [53]. It could be argued, however, that such an “ad lib” prescription better reflects the real world clinical situation and therefore increases the degree to which our results apply to any “real world” breast cancer population (external validity). Finally, we mention the limitation that the original patient number estimation for the KETOCOMP study was based on measuring the absolute change in PA between intervention group 1 and the control group across all three tumor entity cohorts [23]. This resulted in a minimum number of 35 patients needed in each group to detect a 0.3° increase in PA, which might imply that this breast cancer study has been underpowered. However, with the closure of intervention group 1 and focusing on longitudinal body composition changes instead of PA, our analysis was able to detect highly significant effects of a KD consumed during radiotherapy.

## Conclusions

In summary, a KD in a breast cancer population during radiotherapy improved body composition compared to a SD, with reductions in BW that were mostly due to FM loss and a rapid water loss soon after diet onset. The KD also induced some favorable hormonal changes. Importantly, our KD intervention during radiotherapy was well accepted by the women who started it and feasible within our community hospital setting given minimum requirements of a dietician and motivated hospital staff.

## Supplementary information

**Additional file 1: Supplementary Table 1.** Second-order bias-corrected Akaike information criterion (AICc) values for the different models (Eq. 1-6) fitted to the body composition data.

**Additional file 2: Supplementary Table 2.** Regression coefficients and p-values of linear mixed effects models derived from 1000 Monte Carlo simulations of BIA measurements.

**Additional file 3: Supplementary Table 3.** Regression coefficients for linear mixed effects models fitted to the hormone data.

## Data Availability

The datasets used and/or analyzed during the current study are available from the corresponding author on reasonable request.

## Abbreviations

BMI: Body mass index
BW: Body weight
ECW: Extracellular water
FFM: Fat free mass
FM: Fat mass
ICW: Intracellular water
IGF-1: Insulin-like growth factor 1
KD: Ketogenic diet
MAP: Master Amino Acid Pattern
QoL: Qualtiy of Life
PA: Phase angle
PTV: Planning target volume
SD: Standard diet
SMM: Skeletal muscle mass
TBW: Total body water
