# Supplementary material for: Impact of a ketogenic diet intervention during radiotherapy on body composition: III—final results of the KETOCOMP study for breast cancer patients
Source: Breast Cancer Res. 2020 Aug 20;22:94. doi: 10.1186/s13058-020-01331-5 (PMC7441712; doi:10.1186/s13058-020-01331-5)
Supplement: Supplementary file 1 — Additional file 1: Supplementary Table 1. Second-order bias-corrected Akaike information criterion (AICc) values for the different models (Eq. 1-6) fitted to the body composition data. [file 13058_2020_1331_MOESM1_ESM.docx]

**Supplementary Table 1: Second-order bias-corrected Akaike information criterion (AICc) values for the different models (Eq. 1-6) fitted to the body composition data.**

| Model | 1 (basic model) | 2 (+BMI) | 3 (+MAP×time) | 4 (+Age) | 5 (+PTV) | 6 (full model) | Best model without KD variable |
| --- | --- | --- | --- | --- | --- | --- | --- |
| Body weight | 707.1 | 708.5 | 707.5 | **705.2** | 709.1 | 709.2 | 738.8 |
| Fat mass | 608.7 | 610.8 | **607.5** | 608.4 | 608.0 | 609.8 | 638.2 |
| Fat free mass | 742.5 | 744.7 | 744.0 | **740.2** | 744.2 | 745.2 | 753.7 |
| Skeletal muscle mass | 517.6 | 517.4 | 519.1 | **516.6** | 518.8 | 517.5 | 527.5 |
| Phase angle (50kHz) | −156.8 | −155.8 | −155.1 | −161.9 | −154.6 | **−162.7** | **−**155.0 |
| Extracellular water [L] | **319.7** | 321.8 | 321.8 | 321.5 | 320.4 | 325.8 | 331.2 |
| Intracellular water [L] | 391.7 | 392.3 | 392.8 | **388.3** | 393.0 | 389.0 | 394.4 |
| Total body water [L] | 627.6 | 629.2 | 629.4 | **627.6** | 628.5 | 632.0 | 644.8 |

The best model is identified by the smallest AICc value (bold). The last column shows AICc values for the best model re-fitted with omitting the intervention group (KD) variable. BMI: Body mass index; MAP: Master Amino Acid Pattern supplement; PTV: Planning target volume
