# Supplementary material for: Impact of a ketogenic diet intervention during radiotherapy on body composition: III—final results of the KETOCOMP study for breast cancer patients
Source: Breast Cancer Res. 2020 Aug 20;22:94. doi: 10.1186/s13058-020-01331-5 (PMC7441712; doi:10.1186/s13058-020-01331-5)
Supplement: Supplementary file 2 — Additional file 2: Supplementary Table 2. Regression coefficients and p-values of linear mixed effects models derived from 1000 Monte Carlo simulations of BIA measurements. [file 13058_2020_1331_MOESM2_ESM.docx]

**Supplementary Table 2: Regression coefficients and p- values of linear mixed effects models derived from 1000 Monte Carlo simulations of BIA measurements**

|  | **Body weight** | | **Fat mass** | | **Fat free mass** | | **Skeletal muscle mass** | | **Total body water** | |
| --- | --- | --- | --- | --- | --- | --- | --- | --- | --- | --- |
| **Covariate** | **Coefficient** | **p-value** | **Coefficient** | **p-value** | **Coefficient** | **p-value** | **Coefficient** | **p-value** | **Coefficient** | **p-value** |
| Time | 0.04±0.05 kg/week | 0.40 | 0.08±0.11 kg/week | 0.46 | −0.07±0.11 kg/week | 0.51 | −0.01±0.07 kg/week | 0.85 | −0.06±0.08 L/week | 0.45 |
| KD: yes | **−1.56±0.26 kg** | **9.1×10^-10^** | −0.58±0.58 kg | 0.32 | −1.4±0.6 kg | 0.027 | −0.67±0.40 kg | 0.097 | −1.2±0.5 L | 0.0098 |
| Time × KD | **−0.42±0.08 kg/week** | **5.3×10^-7^** | **−0.45±0.17** **kg/week** | **0.0087** | 0.12±0.17 kg/week | 0.47 | −0.01±0.11 kg/week | 0.89 | 0.09±0.12 L/week | 0.45 |
| Baseline BMI | −0.48±0.68 kg/10 kg/m^2^ | 0.48 | **5.4±1.5 kg/10 kg/m^2^** | **3.6×10^-4^** | 0.86±0.77 kg/10 kg/m^2^ | 0.26 | 0.98±0.47 kg/10 kg/m^2^ | 0.037 | 0.69±0.55 L/10 kg/m^2^ | 0.21 |
| Time × MAP | 0.12±0.09 kg/week | 0.19 | 0.13±0.18 kg/week | 0.48 | −0.05±0.16 kg/week | 0.78 | −0.03±0.10 kg/week | 0.80 | −0.02±0.12 L | 0.85 |
| Age | −0.21±0.11 kg/10 years | 0.045 | 0.38±0.22 kg/10 years | 0.087 | −0.36±0.23 kg/10 years | 0.12 | −0.26±0.14 kg/10 years | 0.067 | −0.17±0.16 L/10 years | 0.30 |
| PTV | −0.15±0.23 kg/500ccm | 0.52 | 0.10±0.45 kg/500ccm | 0.82 | 0.60±0.49 kg/500ccm | 0.22 | 0.18±0.29 kg/500ccm | 0.53 | 0.38±0.35 L/500ccm | 0.28 |

To take into account the intra-individual prediction errors in body composition data, 1000 Monte Carlo simulations of a BIA measurement were created for each subject. A new body composition measurement was simulated by drawing a random number with mean located at the actual measurement value and standard deviation equal to 100 g for BW, 1.91 kg for FFM, 1.2 kg for SMM, 0.79 L for ECW and 1.34 L for TBW. A model was fit to each simulated longitudinal body composition dataset and the regression coefficients were averaged. Their standard errors were obtained as an average of the regression coefficient standard errors from each simulation. From the average estimate and its standard error, a two-sided p-value was calculated assuming a Gaussian distribution.
