# Supplementary material for: Impact of a ketogenic diet intervention during radiotherapy on body composition: III—final results of the KETOCOMP study for breast cancer patients
Source: Breast Cancer Res. 2020 Aug 20;22:94. doi: 10.1186/s13058-020-01331-5 (PMC7441712; doi:10.1186/s13058-020-01331-5)
Supplement: Supplementary file 3 — Additional file 3: Supplementary Table 3. Regression coefficients for linear mixed effects models fitted to the hormone data. [file 13058_2020_1331_MOESM3_ESM.docx]

**Supplementary Table 3: Regression coefficients for linear mixed effects models fitted to the hormone data**

|  | Insulin [mU/l] | | IGF-1 [ng/ml] | | | T3 [pg/ml] | |
| --- | --- | --- | --- | --- | --- | --- | --- |
| Covariate | Coefficient | p-value | Coefficient | p-value | Coefficient | | p-value |
| Time | (−0.2±0.2) /week | 0.31 | (−2.8±1.6)/week | 0.074 | (−0.02±0.01) /week | | 0.099 |
| KD: yes | −0.5±1.7 | 0.78 | −0.6±14.1 | 0.97 | −0.07±0.11 | | 0.54 |
| Time × KD | (−0.1±0.2)/week | 0.82 | (−1.5±2.2) /week | 0.51 | **(−0.06±0.01)/week** | | **6.3×10^-5^** |
| Baseline BMI | **(4.5±1.3)/10 kg/m^2^** | **3.9×10^-4^** | (−6.2±11.7)/10 kg/m^2^ | 0.60 | (0.04±0.08)/10 kg/m^2^ | | 0.58 |
| Age | (−0.4±0.6)/10 years | 0.52 | **(−36.8±5.7)/10 years** | **9.7×10^-11^** | (−0.06±0.04)/10 years | | 0.11 |

Regression coefficients estimates are given with their standard error and associated p-value. Note that the average time trend for the SD and KD groups are given by the regression coefficients corresponding to “Time” and “Time” + ”Time × KD”, respectively. BMI: Body mass index; IGF-1: insulin-like growth factor 1, KD: Ketogenic diet
